# Altered Neurochemical Ingredient of Hippocampus in Patients with Bipolar Depression

**DOI:** 10.1155/2012/485249

**Published:** 2012-03-01

**Authors:** Murad Atmaca, Hanefi Yildirim

**Affiliations:** Department of Psychiatry, School of Medicine, Firat University, 23119 Elazig, Turkey

## Abstract

*Background*. In a number of investigations, hippocampal neurochemicals were evaluated in the patients with bipolar disorder who were on their first episode or euthymic periods. However, we did not meet any investigation in which only patients with bipolar depression were examined. As a consequence, the objective of the present study was to examine both sides of hippocampus of patients with bipolar disorder in depressive episode and healthy controls using ^1^H-MRS. *Methods*. Thirteen patients with DSM-IV bipolar I disorder, most recent episode depressed, were recruited from the Department of Psychiatry at Firat University School of Medicine. We also studied 13 healthy comparison subjects who were without any DSM-IV Axis I disorders recruited from the hospital staff. The patients and controls underwent proton magnetic resonance spectroscopy (^1^H-MRS) of their hippocampus. NAA, CHO, and CRE values were measured. *Results*. No significant effect of diagnosis was observed for NAA/CRE ratio. For the NAA/CHO ratio, the ANCOVA with age, gender, and whole brain volume as covariates revealed that the patients with bipolar depression had significantly lower ratio compared to healthy control subjects for right and for left side. As for the CHO/CRE ratio, the difference was statistically significant for right side, with an effect diagnosis of *F* = 4.763, *P* = 0.038, and was very nearly significant for left side, with an effect diagnosis of *F* = 3.732, *P* = 0.064. *Conclusions*. We found that the patients with bipolar depression had lower NAA/CHO and higher CHO/CRE ratios compared to those of healthy control subjects. The findings of the present study also suggest that there may be a degenerative process concerning the hippocampus morphology in the patients with bipolar depression.

## 1. Introduction

Bipolar I disorder is a mood disorder according to Diagnostic and Statistical Manual of Mental Disorders Fourth Version (DSM-IV) characterized by at least one manic or mixed episode. Although the existence of depressive episodes is not required, these type of episodes frequently occurred in bipolar I disorder. Neurobiological factors have been implicated in the pathogenesis of bipolar I disorder, as in other psychiatric disorders. Recently, a huge number of neuroimaging investigations have been performed in patients with various psychiatric disorders to identify morphometric changes in specific brain areas. These techniques are noninvasive ones that provide our best understanding of the connection between the clinical features and related neurobiology in psychiatric disorders including bipolar I disorder. Thus, in patients with bipolar disorder, many investigations have been performed and have revealed some important findings such as decreased subgenual prefrontal cortex volume, decreased prefrontal gray matter, and amygdala volume increase [1–5]. On the other hand, increased white matter hyperintensities and decreased cerebellar areas have been reported [6, 7]. Our group also carried out some structural investigations in different regions of brains of bipolar patients. We performed a volumetric MRI study to assess the subregions of the cingulate gyrus: left anterior cingulate (LAC), left posterior cingulate (LPC), right anterior cingulate (RAC), and right posterior cingulate (RPC) in bipolar patients that were either unmedicated (*n* = 10), on valproate monotherapy (*n* = 10) or on valproate plus quetiapine (*n* = 10) versus healthy comparisons (*n* = 10). In that study, we found that drug-free patients had significantly smaller LAC and LPC volumes compared with valproate and valproate plus quetiapine groups and healthy controls, with a trend toward significant difference between valproate plus quetiapine group and valproate group in regard to only LAC in post hoc comparisons and concluded that valproate and quetiapine might have neuroprotective effects [8]. In our another previous structural MRI investigation, we investigated the corpus callosum (CC) areas by MRI in 12 first-episode patients with bipolar disorder and 12 controls. We found that bipolar patients had significantly smaller areas of total CC, anterior body posterior body, and isthmus compared with healthy control subjects by ANCOVA, with age, gender, and intracranial volume (ICV) as covariates and that there was a negative correlation between total CC, posterior body and isthmus areas, and Young Mania Rating Scale (YMRS) scores, and suggested that the findings suggest that CC morphology may be associated with the pathophysiology of bipolar disorder [9]. As for the functional neuroimaging investigations in bipolar disorder, the published studies alterations in glucose metabolism, regional cerebral blood flow or high-energy phosphate metabolism in patients with bipolar disorder in the prefrontal and temporal cortex, basal ganglia and amygdala [1, 10]. Previously, we performed two-proton magnetic resonance spectroscopy (1H MRS) investigations in bipolar disorder. We first evaluated NAA values in patients with first-episode bipolar disorder and found statistical analysis to reveal a significant effect of diagnosis for NAA/CRE and for NAA/CHO but not for CHO/CRE and concluded that hippocampal neuronal abnormalities might be present at the onset of bipolar I disorder and might be associated with the severity of bipolar I disorder [11]. In our other NAA investigation, we examined the effects of mood stabilizer alone and the combination of mood stabilizer and atypical antipsychotic, quetiapine, on hippocampal neurochemical markers of thirty bipolar disordered patients [12]; ten were first applied patients who never had taken any drug for this condition (drug-free group), ten were on ongoing valproate treatment (valproate group), and the rest were on valproate plus quetiapine treatment (valproate plus quetiapine group). We found that drug-free patients had significantly lower NAA/CRE and NAA/CHO ratios compared with valproate and valproate plus quetiapine groups and healthy controls, with a significant difference between valproate plus quetiapine group and valproate group in regard to only NAA/CHO in post hoc comparisons. Proton magnetic resonance spectroscopy (^1^H-MRS), an increasing trend in psychiatric investigation, allows biochemical constituents to be directly assayed *in vivo*, such as choline-containing compounds (CHO), an index of membrane metabolism, creatine + phosphocreatine (CRE), involved in cell energetic metabolism and *n*-acetyl-containing compounds (especially *N*-acetylaspartate—NAA). While CHO and CRE are present in neurons and in glial cells, NAA is found primarily in neurons [13] and in highest concentrations in pyramidal glutamatergic neurons [14]. NAA, a representative marker of neuronal structural integrity, seems sensitive to mitochondrial oxidative phosphorylation and it may correlate highly with tissue glutamate levels [15, 16]. Low NAA is thought to represent loss of neurons and/or axons, reduction of interneuronal neuropil, and neuronal or axonal metabolic dysfunction or damage [17, 18]. ^1^H MRS studies concerning bipolar disorder focused on dorsolateral prefrontal cortex (DLPFC) and hippocampal regions. A ^1^H MRS study showed significant reductions of NAA peaks in the DLPFC of adult bipolar disorder subjects [19], whereas two other MRS studies [20, 21] did not find any differences in DLPFC or frontal lobes. Chang et al. [22] reported reduced NAA levels in DLPFC in a sample of pediatric bipolar patients who had a parent with bipolar disorder. Furthermore, there is extensive literature from functional imaging and postmortem studies in support of DLPFC dysfunction in bipolar disorder [23, 24]. Studies using high-resolution MRS reveal that unmedicated patients with bipolar disorder have decreased levels bilaterally of NAA in the hippocampus [21], as compared with healthy control subjects. Moreover, therapeutic doses of lithium reverse these decreased levels of NAA in their brain [25]. Deicken et al. [26] found low NAA bilaterally in the absence of smaller hippocampal volume as measured by MRI, supporting the idea that NAA might be a more sensitive marker of neuronal damage or loss than quantitative MRI measurements of tissue loss. In a number of investigations, hipopcampal neurochemicals were evaluated in the patients with bipolar disorder who were on their first episode or euthymic periods. However, we did not meet any investigation in which only patients with bipolar depression were examined. As a consequence, the objective of the present study was to examine both sides of hippocampus of patients with bipolar disorder in depressive episode and healthy controls using ^1^H-MRS.

## 2. Methods

### 2.1. Subjects and Clinical Evaluation

 Sixteen patients with DSM-IV bipolar I disorder, most recent episode depressed who suffered from MRI for differential diagnosis, were recruited from Department of Psychiatry at Firat University School of Medicine. We also studied 16 healthy comparison subjects who were without any DSM-IV Axis I disorders recruited from the hospital staff. All subjects participated after reviewing and signing the consent form. The Diagnostic and Statistical Manual of Mental Disorders Fourth Version (DSM-IV) diagnoses were obtained using Turkish version of Structured Clinical Interview for DSM-IV (SCID) [27].

 Exclusion criteria were the presence of any comorbid psychiatric disorder, current serious medical problems, or alcohol/substance abuse within the 6 months preceding the study. Of the patients, two had borderline personality disorder, one had obsessive compulsive personality disorder, and one had dependent personality traits. Healthy control subjects had no DSM-IV Axis I disorders in self or in a first-degree relative, as determined by the SCID nonpatient version, no current medical problems, neurologic or psychiatric histories, and no use of psychoactive medication within 2 weeks of the study.

 The severity of depression and manic symptoms was evaluated by using the Hamilton Depression rating Scale (HDRS) [28] and Young Mania Rating Scale (YMRS) [29], respectively.

### 2.2. MRI Procedure

 Multiple-slice ^1^H-MRSI was performed on a conventional GE-SIGNA 1.5-Tesla MR imaging system (GE Medical Systems Milwaukee, WI). A high-resolution structural image of the entire brain was obtained using sagittally acquired 3D spiral fast spin echo high-resolution images (repetition time (TR) = 2000 ms, echo time (TE) = 15.6 ms, field of view (FOV) = 240 mm, flip angle = 20°, bandwidth = 20.8, slice thickness = 2.4 mm, echo spacing = 15.6 ms, 8 echoes, matrix size = 240, resolution = 0.9375 × 0.9375 × 2.4 mm). The hippocampal region was drawn with reference to standard anatomic atlas [30]. Anatomic measurements were obtained on a computer advanced workstation with the GE Volume Viewer voxtool 4.2 program. Each voxel had nominal dimensions of 10 mm × 10 mm × 2.4 mm (0.24 mL) with an actual volume of 0.4 mL, based on full width at half maximum (FWHM).

**Figure 1:**
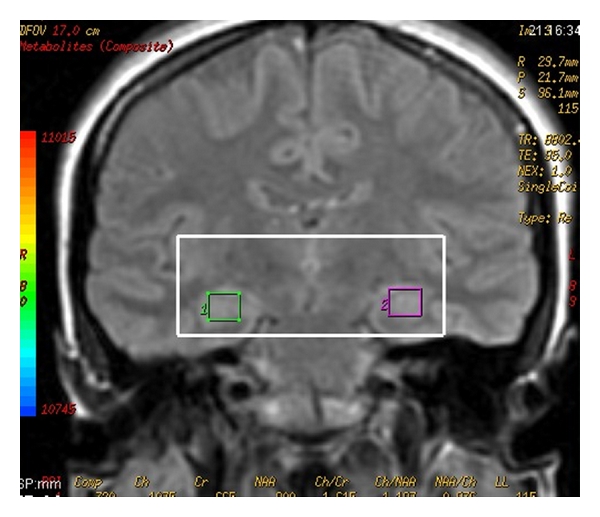
Position of hippocampal voxels and sample magnetic resonance spectrum.

 In the present study, as neurochemicals, it was examined NAA, CRE, and CHO. The peak levels for these neurochemicals were detected automatically for all voxels. The signal strength around the NAA, CHO, and CRE signal positions was integrated to produce three 18 × 18 arrays of metabolite signals. Position of hippocampal voxels and sample magnetic resonance spectrum are presented in Figure 1. Meanwhile, to minimize errors caused by changes in magnetic field homogeneity and tissue volume, CRE signals were designated as the reference, with the results presented as metabolite NAA CHO to CRE ratio. On the other hand, to account for possible alterations in the CRE peak as an internal reference, NAA metabolite measurements are additionally reported as NAA/CHO ratios [31].

### 2.3. Statistical Analysis

 Statistical analyses were conducted using SPSS for Windows software, version 10.0 (SPSS, Chicago, IL). Group differences in demographic variables involving continuous data were computed using independent *t*-test. Between-group comparisons involving categorical data were assessed using chi-square test. All the metabolite measurements were found to be normally distributed. For each metabolite level and metabolite ratio differences between patients and controls were tested separately by a one-way analysis of covariance (ANCOVA), with hemisphere (left or right) as the within-group factor and diagnosis as the between-group factor. Post hoc analyses were performed by Tukey's honestly significant difference test. Correlations were assessed with Pearson's correlation test.

## 3. Results

 The main demographic and clinical features of the sample are summarized in Table 1. There were no significant differences between the two groups for age, whole brain volume, gray or white matter volumes (independent sample *t*-test, *P* > 0.05), gender, or handedness (chi-square, *P* > 0.05).

 ANCOVA with age, gender, and whole brain volume as covariates demonstrated that patients with bipolar disorder, at most recent depressive episode, did not have different NAA/CRE ratio compared to that of healthy controls for both right (effect of diagnosis; *F* = 2.490, *P* = 0.127; mean ratio; 1.38 ± 0.25 for patients and 1.26 ± 0.25 for controls) and left sides (effect of diagnosis; *F* = 2.326, *P* = 0.139; mean ratio; 1.30 ± 0.42 for patients and 1.34 ± 0.18 for controls) even when the two groups were stratified for gender (ANCOVA with age and ICV as covariates, *P* > 0.05). On the other hand, no significant effect of diagnosis was observed for NAA/CRE ratio (*F* = 0.073, *P* = 0789; mean ratio; 1.32 ± 0.25 for patients 1.29 ± 0.15 for controls). For the NAA/CHO ratio, the ANCOVA with age, gender, and whole brain volume as covariates revealed that the patients with bipolar depression had significantly lower ratio compared to healthy control subjects for right (effect of diagnosis; *F* = 5.105, *P* = 0.032; mean ratio; 0.78 ± 0.36 for patients and 0.94 ± 0.17 for controls) and for left sides (effect of diagnosis; *F* = 4.724, *P* = 0.043; mean ratio; 0.76 ± 0.27 for patients and 0.97 ± 0.15 for controls). With regard to total NAA/CHO, the patients had lower ratio (effect of diagnosis; *F* = 5.251, *P* = 0.030; mean ratio; 0.74 ± 0.21 for patients and 0.95 ± 0.14 for controls). As for the CHO/CRE ratio, the difference was statistically significant for right side, with an effect diagnosis of *F* = 4.763, *P* = 0.038 (mean ratio; 1.99 ± 0.63 for patients and 1.36 ± 0.25 for controls), and was very nearly significant for left side, with an effect diagnosis of *F* = 3.732, *P* = 0.064 (mean ratio; 1.85 ± 0.84 for patients and 1.41 ± 0.26 for controls). With regard to total CHO/CRE ratio, the effect of diagnosis was considerable (*F* = 7.921, *P* = 0.009, mean ratio 1.87 ± 0.54 for patients and 1.37 ± 0.19 for controls). Following significant and nearly significant correlations were determined: NAA/CHO and YMRS (*r* = 0.55, *P* = 0.028), CHO/CRE and YMRS (*r* = −0.48, *P* = 0.061), NAA/CHO and age (*r* = −0.70, *P* = 0.002), CHO/CRE and age (*r* = 0.69, *P* = 0.003). The Pearson's correlation test could not reach statistical significance for any relation between NAA/CRE, NAA/CHO, or CHO/CRE ratio of the hippocampus, years of education, age of onset, or total scores of the HDRS.

## 4. Discussion

The main findings of the present study were as follows. (i) No significant effect of diagnosis was observed for NAA/CRE ratio. (ii) For the NAA/CHO ratio, the ANCOVA with age, gender, and whole brain volume as covariates revealed that the patients with bipolar depression had significantly lower ratio compared to healthy control subjects for right and for left side. With regard to total NAA/CHO, the patients had lower ratio. (iii) As for the CHO/CRE ratio, the difference was statistically significant for right side, with an effect diagnosis of *F* = 4.763, *P* = 0.038, and was very nearly significant for left side, with a effect diagnosis of *F* = 3.732, *P* = 0.064. With regard to total CHO/CRE ratio, the effect of diagnosis was considerable. (iv) Following significant and nearly significant correlations were determined: NAA/CHO and YMRS (*r* = 0.55, *P* = 0.028), CHO/CRE and YMRS (*r* = −0.48, *P* = 0.061), NAA/CHO and age (*r* = −0.70, *P* = 0.002), CHO/CRE and age (*r* = 0.69, *P* = 0.003). First of all, our findings reveal that NAA/CHO values were lower in patients with panic disorder compared to healthy controls for both sides of the hippocampus and there were significant relationships between NAA/CHO and YMRS (*r* = 0.55, *P* = 0.028) and NAA/CHO and age (*r* = −0.70, *P* = 0.002) led us to consider that reduced neuronal density or even a neurodegeneration of the hippocampal region might exist, because NAA is considered as a measure of neuronal integrity, as discussed in our other investigation on obsessive compulsive disorder, another anxiety disorder [32]. On the other hand, we previously performed two proton magnetic resonance spectroscopy (^1^H MRS) investigations in bipolar disorder. We first evaluated NAA values in patients with first-episode bipolar disorder and found statistical analysis to reveal a significant effect of diagnosis for NAA/CRE and for NAA/CHO but not for CHO/CRE and concluded that hippocampal neuronal abnormalities might be present at the onset of bipolar I disorder and might be associated with the severity of bipolar I disorder [11]. In this context, the existence of diagnostic effect for NAA/CRE and NAA/CHO but not for CHO/CRE may suggest that changes in NAA seem to be associated with both sides of the bipolar disorder, that is, manic and depressive dimensions. In our another NAA investigation on bipolar disorder, we examined the effects of valproate, a mood stabilizer, alone, and the combination of valproate and atypical antipsychotic, quetiapine, on hippocampal neurochemical markers of thirty bipolar disordered patients [12]; ten were first applied patients who never had taken any drug for this condition (drug-free group), ten were ongoing valproate treatment (valproate group), and the rest were on valproate plus quetiapine treatment (valproate plus quetiapine group). We detected that drug-free patients had significantly lower NAA/CRE and NAA/CHO ratios compared with valproate and valproate plus quetiapine groups and healthy controls, with a significant difference between valproate plus quetiapine group and valproate group in regard to only NAA/CHO in post hoc comparisons. This second investigation supports the speculation that reduced neuronal density or even a neurodegeneration of the hippocampal region might exist in bipolar disoder itself, independent of its period. Moreover, significantly negative correlation between the NAA/CHO ratio and age further supports the notion indicating that there may be a neurodegenerative process. As for our second important finding for CHO/CRE ratio, the difference was statistically significant for right side, with an effect diagnosis of *F* = 4.763, *P* = 0.038, and was very nearly significant for left side, with a effect diagnosis of *F* = 3.732, *P* = 0.064. CHO is an important neurochemical in the pathogenesis of neuropsychiatric disorders, particularly including mood disorders. Choline is often released under pathological conditions from its stores in cell membranes and is therefore considered to be a marker of cellular membrane turnover and active neurodegeneration [33, 34]. It is accepted as a metabolic marker of membrane density and integrity, that is, phospholipid synthesis and degradation [35]. Any neuropathological process leading to cell membrane breakdown causes to release CHO and rises the free CHO, contributing to an increased cellular membrane turnover and active neurodegeneration [36]. At this point, it should be mentioned that circulating corticosteroids have an important role on the modulation of cholinergic activity in the hippocampal region [37]. Furthermore, these neurohormones seem to increase the vulnerability of the septo-hippocampal cholinergic neurons to noxious insult [38]. So, we speculate that CHO levels detected in the patients with bipolar depression may reflect stress reactive remodeling processes, as also suggested by Andrea et al. [37].

Some limitations of the present study must be ackonwledged. First, actually, as with all neuroimaging modalities, the number size of the groups enrolled in a given study tends to be small. However, it should be noted that the small sample size is limited to make conclusive results. Second, no control for partial volume effect was another limitation. Third, possible long-lasting effects of various medications on the hippocampus may have affected our findings. Finally, only the hippocampus was investigated in this study; therefore we do not know exactly but it is possible that these findings are not spesific for the hippocampus and that this finding may be generalized to the other brain regions as well. However, to access strong evidence, further investigation with more patients and in multiple brain regions is needed.

In conclusion, we found that the patients with bipolar depression had lower NAA/CHO and higher CHO/CRE ratios compared to those of healthy control subjects. The findings of the present study also suggest that there may be a degenerative process concerning the hippocampus morphology in the patients with bipolar depression.

## Figures and Tables

**Table 1:** Clinical and demographic information for healthy subjects and patients with bipolar depression.

	Patients (*n* = 16)	Controls (*n* = 16)	Effect of diagnosis
Age	28.1 ± 3.4	30.6 ± 4.2	
Gender (F/M)	4/12	6/10	
Graduated from high school	12	14	
Handedness (right)	16	16	
Number of subjects who had family history	2	—	
YMRS score	6.13 ± 1.93	—	
HDRS score	22.06 ± 4.36	5.88 ± 2.06	
NAA			
Right	593.63 ± 412.09	838.56 ± 176.19	
Left	633.69 ± 386.88	899.19 ± 125.39	
CHO			
Right	793.31 ± 430.69	898.88 ± 139.14	
Left	806.37 ± 361.29	944.37 ± 144.94	
CRE			
Right	420.63 ± 253.52	669.32 ± 89.81	
Left	486.19 ± 281.01	684.50 ± 132.28	
NAA/CRE	1.32 ± 0.25	1.29 ± 0.15	0.073
Right	1.38 ± 0.25	1.26 ± 0.25	2.490
Left	1.30 ± 0.42	1.34 ± 0.18	2.326
NAA/CHO	0.74 ± 0.21	0.95 ± 0.14*	5.251
Right	0.78 ± 0.36	0.94 ± 0.17*	5.105
Left	0.76 ± 0.27	0.97 ± 0.15	4.724
CHO/CRE	1.87 ± 0.54	1.37 ± 0.19**	7.921
Right	1.99 ± 0.63	1.36 ± 0.25*	4.763
Left	1.85 ± 0.84	1.41 ± 0.26***	3.732

Statistically significant comparisons were indicated with an asterisk.

ICV: Intracranial volume; Y-BOCS: Yale Brown obsession compulsion scale: NAA, N-acetyl aspartate; CHO: choline; CRE: creatine.

*P < 0.05, **P < 0.01, ***P = 0.064.
